# Social Context Modulates Tolerance for Pragmatic Violations in Binary but Not Graded Judgments

**DOI:** 10.3389/fpsyg.2019.00510

**Published:** 2019-03-20

**Authors:** Les Sikos, Minjae Kim, Daniel J. Grodner

**Affiliations:** ^1^ Department of Language Science and Technology, Saarland University, Saarbrücken, Germany; ^2^ Department of Psychology, Boston College, Boston, MA, United States; ^3^ Department of Psychology, Swarthmore College, Swarthmore, PA, United States

**Keywords:** language, pragmatics, inference, pragmatic tolerance, scalar implicature, truth value judgment, social cognition

## Abstract

A common method for investigating pragmatic processing and its development in children is to have participants make binary judgments of underinformative (UI) statements such as *Some elephants are mammals*. Rejection of such statements indicates that a (not-all) scalar implicature has been computed. Acceptance of UI statements is typically taken as evidence that the perceiver has not computed an implicature. Under this assumption, the results of binary judgment studies in children and adults suggest that computing an implicature may be cognitively costly. For instance, children under 7 years of age are systematically more likely to accept UI statements compared to adults. This makes sense if children have fewer processing resources than adults. However, Katsos and Bishop (2011) found that young children are able to detect violations of informativeness when given graded rather than binary response options. They propose that children simply have a greater tolerance for pragmatic violations than do adults. The present work examines whether this pragmatic tolerance plays a role in adult binary judgment tasks. We manipulated social attributes of a speaker in an attempt to influence how accepting a perceiver might be of the speaker’s utterances. This manipulation affected acceptability rates for binary judgments (Experiment 1) but not for graded judgments (Experiment 2). These results raise concerns about the widespread use of binary choice tasks for investigating pragmatic processing and undermine the existing evidence suggesting that computing scalar implicatures is costly.

## Introduction

Much of what we communicate in conversation is implicit. For example, if a speaker says, “Some of the students passed the test,” comprehenders often infer that *not all* of the students passed. This is a pragmatic inference. It arises because communication is typically cooperative. Cooperative speakers should, among other things, make the strongest statement compatible with their knowledge. This follows from the maxim of quantity (Grice, 1975). The speaker chose a relatively vague expression (*some*) rather than a more specific one (*all*). The comprehender can thus infer that the speaker was not in a position to use the more informative expression. This frequently leads to the inference that a stronger statement (*All of the students passed the test*) is false.

This is an example of a *scalar implicature* (Horn, 1972). In recent years, scalar implicatures have become a central testing ground for investigating how implicit meanings are computed and how pragmatic communication abilities develop. To explore these issues, researchers frequently ask participants to judge underinformative (UI) statements such as *Some elephants are mammals* (see Katsos and Cummins, 2012 for a review). These utterances are literally true, but their implicit *not-all* meaning is false. The rejection of a UI statement in a binary sentence acceptability judgment task is thought to indicate that a pragmatic inference has been computed. Acceptance is taken as an indication that only a literal interpretation has been computed.

There is considerable variation across individuals and situations in judgments for UI statements. Studies report that anywhere from 23 to 83% of adult respondents judge such sentences false depending on various factors (see Dieussaert et al., 2011 for review). One important factor appears to be cognitive processing resources. Participants take longer to judge UI statements as false rather than true. This is consistent with the notion that participants initially compute the literal meaning of UIs before engaging in an effortful second stage process of computing the pragmatic meaning. In support, when participants are given less time to respond (Bott and Noveck, 2004; Bott et al., 2012) or are asked to do a secondary memory task (De Neys and Schaeken, 2007; Dieussaert et al., 2011; Marty and Chemla, 2013) the acceptance rate of UI statements increases, but not the acceptance rates for patently true or false statements (e.g., *All elephants are mammals, Some elephants are reptiles*). Further, individuals with smaller working memory capacity exhibit greater acceptance of UI sentences (Feeney et al., 2004; Dieussaert et al., 2011). Acceptance rates also decrease when a larger proportion of stimuli are UI statements or when alternative utterances are made more salient (Foppolo et al., 2012). Both of these manipulations should make it easier to make the comparisons necessary to generate the inference. These results are anticipated if computing scalar inferences requires time and cognitive resources.

In contrast to adult response patterns, developmental studies on the acquisition of scalar inference report that children under 7-years-old reliably accept UI statements.^1^ This has led many researchers to conclude that young children lack the cognitive resources or the pragmatic competence to derive conversational inferences at adult-like levels (see Noveck and Reboul, 2008). However, studies that do not use judgment tasks generally indicate that young children can generate scalar implicatures. Pouscoulous et al. (2007) asked children to perform an act out task to make a display of boxes accurately conform to a statement. In a situation where five of five boxes contained a token, the experimenter said, “I would like some of the boxes to contain a token.” Nearly 70% of 4-year-olds removed a coin from at least one of the boxes. This strongly suggests that they generated a *not all* implicature. Similar evidence was found by (Horowitz et al., 2018; Experiment 2) using a referential identification task. The experimenter said, “On the cover of my book, some of the pictures are cats.” Children as young as 4.5 years old reliably selected a book for which two of four pictures were cats more often than a book for which four of four pictures were cats.

Katsos and Bishop (2011; see also Veenstra et al., 2018) propose that the acceptance of pragmatically infelicitous statements in binary judgment tasks may reflect a greater tolerance of pragmatic violations rather than a lack of pragmatic competence *per se*. They found that when participants were given a ternary rather than binary judgment task (awarding a cartoon speaker a “small,” “big,” or “huge” strawberry reward), 5- to 6-year-old children and adults were both sensitive to informativity (i.e., they gave UI statements a smaller reward than optimally informative statements such as *Some mammals are elephants*) and tolerant of pragmatic violations (i.e., they gave UI statements a bigger reward than false statements). In a separate study, they replicated the typical pattern whereby children at this age systematically accept UI statements in a binary judgment task. Katsos and Bishop concluded that children do in fact detect violations of informativity for UI statements, but do not consider these pragmatic violations grave enough to warrant outright rejection in a binary choice task. In other words, children may in general be more charitable and forgiving in binary judgment tasks than adults.

Note that recognizing UI statements as underinformative requires computing alternative statements that might have been made (such as *All elephants are mammals*) and determining whether any of these alternatives are more optimally informative than what was actually said. These are precisely the steps involved in generating a scalar implicature. Indeed, the computation of alternatives has been proposed as the primary cognitive bottleneck in scalar implicature computation in adults and children (Barner et al., 2011; Marty and Chemla, 2013; Tiel and Schaeken, 2017). Katsos and Bishop’s pattern of results indicates that children do generate scalar inferences and that this is observable when provided with an appropriate response scale. This result is thus problematic for the view that children lack the cognitive resources or pragmatic skills necessary to generate scalar implicatures. It also calls into question the use of binary choice scales for investigating scalar implicatures in children. The primary goal of the current studies is to examine whether pragmatic tolerance might also play a role in binary judgment tasks for adults.

A potential issue with binary response options is that they artificially constrain the perceivers’ choices. In natural conversation, there are many moves available to an interlocutor who is asked to judge the validity of a statement. For instance, a UI statement might elicit an explanatory qualification (*True, but incomplete or inappropriate; Not quite*) or a request for clarification (*Do you mean not all?*). Indeed in most circumstances, it would be uncooperative to merely tell the speaker that they were right or wrong without providing some additional feedback. This is because there are multiple ways that a statement can be infelicitous. It may be false, off topic, vague, suffer from presupposition failure (e.g., *The current king of France is bald*), or otherwise inapt. A UI statement is neither completely true nor false but pragmatically odd. Thus, even when an individual computes the scalar inference, making a binary judgment compels the perceiver to make a complex metalinguistic judgment about where to place the threshold for acceptability. This raises the possibility that variability in binary response tasks reflects differences in response selection processes when faced with two poor options rather than, or in addition to, differences in computing a pragmatic inference. On this view, we would anticipate that determining where to set the threshold in a binary choice task could be influenced by factors that affect how forgiving the addressee might be toward the speaker’s utterance. This would be true even in cases where these factors are not directly relevant to whether an implicature has been generated.

In contrast, a ternary judgment task provides an intermediate response option that allows respondents an explicit way to signal that UI statements are worse than patently true statements, but better than patently false ones. If so, in situations where participants are provided with three response options rather than two, the intermediate response should be favored (ala Katsos and Bishop, 2011) regardless of the social context or cognitive task demands.

Most previous studies of adult UI sentence processing have asked participants to make judgments on isolated, context-free sentences as stimuli. However, computing a pragmatic inference requires that the comprehender recover the communicative intentions of a cooperative speaker. With context-free sentences, it is unclear what the communicative intentions of the speaker might be: some participants may not attempt to compute a pragmatic interpretation at all given the lack of social context, while others might attempt to attribute particular characteristics and intentions to the speaker in order to judge their pragmatic felicity. As a result, variability in response judgments could be at least partially due to differences in the social attributions that comprehenders covertly ascribe to the disembodied speaker. In an attempt to control this potential aspect of variability, the studies below provide rich communicative contexts with clear goals within which participants are asked to make their judgments.

Furthermore, we hypothesized that social attributes of the speaker might influence how tolerant the perceiver is of the speaker’s utterance. For example, people may be more tolerant of pragmatic violations from speakers they consider to be more likeable. While such attributes do not change the fundamental communicative task and hence should not affect whether an implicature has been drawn, they may make the participant more or less accepting of the speaker’s utterances. The experiments below directly test this hypothesis by manipulating the social attributes of the speaker. If variability in binary choice tasks reflects response selection processes rather than different rates of implicature computation, then this social manipulation will have a greater effect on judgments of UI statements when using a binary scale (Experiment 1) than when using a ternary scale (Experiment 2). In sum, we are interested in whether pragmatic tolerance is affected by social attributes of the speaker, a manipulation that should not directly affect implicature computation *per se*.

## Experiment 1

The goal of Experiment 1 was to test whether attributes of the speaker that are not directly related to the communicative task can affect adult comprehenders’ tolerance for pragmatic violations in a binary judgment task. Participants were provided with a specific social context. They were assigned to tutor an 8-year-old boy on a biology exam on which he was asked to create quantified statements involving animal species and classes. This task provides a plausible cover story for why the speaker might make UI and patently false statements. It also makes clear the purpose of his utterances and the perceiver’s role in the communication. Participants were given a brief description of the student as a Sympathetic, Unsympathetic, or Non-native English-speaking child. The Sympathetic speaker was described as kind and adorable. The Unsympathetic speaker was depicted as cruel and obnoxious. The Non-native speaker was described as speaking English as a foreign language. Importantly, his native language was described as lacking quantifiers.

The aim of this speaker manipulation was to create differing social contexts that might influence adults into being more or less charitable with their judgments of the speaker’s pragmatic violations. For instance, previous work has shown that individuals who are perceived as more likeable receive higher scores on performance assessments in various situations (e.g., Sonnentag, 1998). It was expected that the Sympathetic speaker condition would elicit greater charitability from participants. This in turn might engender increased tolerance for pragmatic infelicity relative to the Unsympathetic condition. The Non-native speaker was included to potentially increase the rate of rejections by providing social motivation to focus specifically on the appropriate use of quantifiers. Since participants were told that Bobby’s native tongue lacks words for specifying quantities, they may have elected to pay special attention to his use of quantifiers in order to help him. This could have led to decreased tolerance for using *some* when *all* would have been more informative compared with the other speaker conditions.

Though speaker type was manipulated between subjects by altering the introductory text, the stimuli, feedback options, and core judgment task were identical for all participants. UI statements in this test-taking context are less optimally informative than a potential alternative statement for all three speaker types. Thus, we should anticipate that implicature rates are similar across the different speakers. If the rate of rejections is different across speakers, this would be evidence that binary judgments are driven by processes other than implicature calculation *per se*.

### Materials and Methods

#### Participants

A total of 102 English-speaking adults were recruited to participate in an online questionnaire through Amazon’s Mechanical Turk in exchange for $0.60. Participants were restricted to those living in the United States, who had completed at least 100 Human Intelligence Tasks (HITs), and who had an excellent performance record on previous HITs (minimum 97% approval rating). The survey was implemented and hosted on Qualtrics. Four participants failed to submit their data at the end of the survey.

#### Stimuli

A total of 120 categorical statements were constructed in 6 sentence types, with 20 statements per type (Table 1). All statements contained a quantifier (*all* or *some*) followed by a subset-superset relationship that paired an animal exemplar (subset) with an animal category (superset). Critical items (UI) were literally true but pragmatically false. Thus, acceptability judgments for such items had no correct or incorrect answer. The remaining sentence types were fillers that described either patently true or patently false subset-superset relations. Ten counterbalanced lists were constructed from these materials such that each list contained ten UI items and ten filler items (two items each of sentence types F1–F5), and no exemplar from a category was used more than once per list. Thus, each list contained 50% UI statements. This proportion has been shown to elicit a high percentage of pragmatic responses in adults (Dieussaert et al., 2011).

**Table 1:** Examples of sentence types.

Type	Example	Correct response
F1	All birds are parrots	“Not quite”
F2	All cats are birds	“Not quite”
F3	All parrots are birds	“That’s right”
F4	Some birds are parrots	“That’s right”
F5	Some cats are birds	“Not quite”
**UI**	**Some parrots are birds**	**?**

#### Instructions

Three parallel sets of instructions were created. They differed only in their characterization of the speaker. All participants saw the following: “Imagine that you have been assigned as a tutor to a young student named Bobby. Bobby is currently studying basic biology. He has just taken a test in which he had to make true sentences out of animal names, animal traits and amount words (‘some,’ ‘all,’ ‘none’). While he has a solid understanding of the animals he studied in class, he has trouble forming appropriate sentences to communicate his knowledge. Your task is to go over each item of the test with Bobby, tell him how he did, and to provide additional feedback to help him create better sentences.” Participants then read one of the following descriptions:

1. *Sympathetic speaker*. “Bobby’s teacher has told you that Bobby is an adorable, funny, outgoing, 8-year-old boy with an unfortunate developmental disorder. Like most children with this disorder, Bobby is eager to interact socially with the people around him but he is hindered with significant speech and language delays. Although Bobby is now a reasonably good communicator, he still lags significantly behind his age-matched peers.”
2. *Unsympathetic speaker*. “Bobby’s teacher has told you that Bobby is a very difficult and obnoxious 8-year-old boy who is often suspended from school because of his repeated violent outbursts. For example, he recently broke a 5-year-old girl’s arm and then laughed at her while she cried. His teachers have told you that Bobby learns best when given clear and direct feedback on tests and assignments.”
3. *Non-native speaker*. “Bobby’s teacher has told you that Bobby is a bright, friendly, 8-year-old boy from Brazil who speaks Gazuungu, an Amazonian language that is known for a number of unusual features. In particular, Gazuungu has no ‘amount words’ for generic quantities less than 10, so it has no equivalents for English words like ‘some.’ Instead, quantities less than 10 must be described using exact numbers. Bobby already knows quite a bit of English but he would like to learn to speak it perfectly. Bobby is patient and does not mind being corrected because it means he is learning.”

#### Procedure

Participants were randomly assigned to a speaker condition. After the instructions, participants completed two practice items (not UI statements). Participants were then randomly assigned to one of the 10 stimulus lists. All 20 experimental items were presented on a single screen with the order of items randomized for each participant. Participants responded to each item by selecting between two radio buttons labeled “That’s right” and “Not quite,” and then provided any additional explanation they thought might be useful for Bobby in a text entry field (e.g., “That’s right. Tigers, like other mammals, have fur”). The survey took approximately 10–15 min to complete.

##### Exit Survey

Following the experimental task, participants were given three 3-option multiple choice questions designed to assess attentiveness to the speaker characteristics: (1) *How old is Bobby?* Options: 6, 8, 12; (2) *How was this student described?* Options: Kind, Amazonian, Obnoxious; and (3) *What subject is he studying?* Options: Biology, Mathematics, Geography. Participants were then asked to judge how likeable Bobby was on a 7-point Likert scale followed by eight demographic questions.

### Results

#### Statistical Methods and Exclusion Criteria

Response data were modeled with logistic mixed effect regression using the glmer function in the lme4 package within the statistical language R (Bates et al., 2014b) and all models consisted of the maximal participant and item random effects structure justified by the data and design (Barr et al., 2013; Bates et al., 2014a). To render model coefficients more interpretable, continuous independent variables were centered around their mean and categorically manipulated predictors were sum coded. Reported coefficients are in logit units.

**Figure 1:**
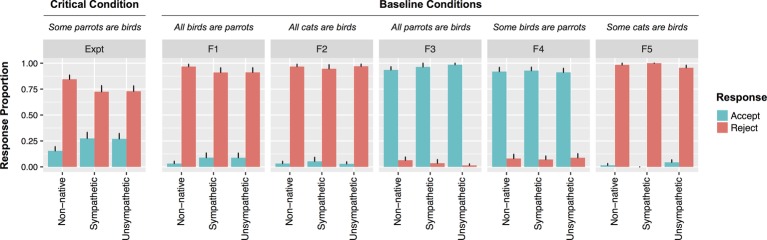
Responses to all statement types by speaker condition from Experiment 1. Error bars indicate standard errors.

Two participants were eliminated for reporting that their age of English acquisition was in adulthood (Each learned at 24 or older, all other participants learned at age 6 or younger). The mean accuracy for responses to filler items (statements type F1-F5) was used as a proxy for attentiveness to the task. Three participants were excluded for accuracy rates below 70%. The remaining 93 participants were relatively evenly distributed across speaker conditions (*N_Non-native_* = 31; *N_Sympathetic_* = 28; *N_Unsympathetic_* = 34). For these participants, mean accuracy rates to filler items were high (*M =* 95%, *SE =* 8.3%) and did not differ across conditions (*z*s ≪ 1). Responses are depicted in Figure 1.

#### Judgments of UI Statements

For UI sentences, the rate of rejections was reliably affected by speaker type: A maximum likelihood ratio test revealed that a model containing speaker type as a fixed effect provided a better fit to the data than one without (*χ2*[2] = 5.15, *p* = 0.076). Pairwise comparisons indicated that Non-native Bobby was reliably more likely to be rejected than Unsympathetic Bobby (*β* = 1.93, *SE* = 0.98, *z* = 1.97, *p* < 0.05) and marginally more than Sympathetic Bobby (*β* = 1.89, *SE* = 1.01, *z* = 1.88, *p* = 0.06). There were no differences in rejections for Sympathetic and Unsympathetic Bobby (*z =* 0.09). There were no effects of speaker condition for any of the filler sentence categories (all *z*s < 1).

#### Exit Survey Results

Participants were extremely accurate at providing Bobby’s age (93.9%), and academic subject (98%). Performance was not significantly different across conditions (*t*s < 1). However, performance was less impressive for remembering the critical description of Bobby (79.8%). Only 50% of participants in the Unsympathetic condition selected “obnoxious” as the best description of Bobby, while the remaining 50% selected “kind.” In contrast, 100 and 97% of participants in the Sympathetic and Non-native speaker conditions respectively selected the appropriate descriptor. It was important to establish that the effect of speaker type on UI judgments was driven by participants who paid attention to the description. To this end, analyses were repeated excluding individuals who provided the wrong description for Bobby. When only responders who were attending to the key manipulation were considered, the trends observed for the whole data set strengthened. A model containing speaker type as a fixed effect provided a reliably better fit to the data than one without (*χ2*[2] = 6.6, *p* < 0.05). Pairwise comparisons indicated that Non-native Bobby was significantly more likely to be rejected than either Sympathetic Bobby (*β* = 2.83, *SE* = 1.42, *z* = 1.99, *p* < 0.05) or Unsympathetic Bobby (*β* = 2.31, *SE* = 1.02, *z* = 2.26, *p* < 0.05). There were no differences in rejections for Sympathetic and Unsympathetic Bobby (*z* < 1). There were no effects of speaker condition for any of the filler sentence categories (all *z*s < 1).

#### Likeability

Surprisingly, participants in the Non-native speaker condition rated Bobby significantly less likeable than those in either the Unsympathetic (*F*(1,65) = 265, *p* < 0.001) or Sympathetic (*F*(1,57) = 249, *p* < 0.001) speaker conditions (Non-native: *M* = 2.24, *SE* = 0.19; Unsympathetic: *M* = 6.0, *SE* = 0.16; Sympathetic: *M* = 6.21, *SE* = 0.17). These differences persisted when only participants who correctly recalled the speaker description were included in the analysis (*ps* < 0.001; Non-native: *M* = 2.16, *SE* = 0.17; Unsympathetic: *M* = 5.83, *SE* = 0.19; Sympathetic: *M* = 6.21, *SE* = 0.17). It was unexpected to find that Non-native Bobby was perceived to be the least likeable and that Unsympathetic Bobby was rated nearly as likeable as Sympathetic Bobby. We discuss possible explanations for this below.

**Figure 2:**
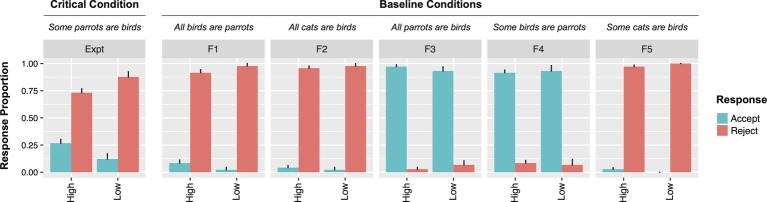
Responses by Low (1–2) versus High (3–7) speaker likeability from Experiment 1. Note that all analyses in the text were performed with likeability as a (non-dichotomized) continuous variable.

A mixed effects model with likeability as a predictor of rejections fared reliably better than one without (*χ2*[2] = 4.3; *p <* 0.05). The more likeable participants rated Bobby, the less likely they were to reject UI statements (*β* = 0.37, *SE* = 0.18, *z* = 2, *p* < 0.05). When only participants who accurately recalled the description of Bobby were included, the relationship between likeability and rejection rate was still present (*χ2*[2] = 6.2; *β* = 0.53, *SE* = 0.23, *z* = 2.33, *p* < 0.05). In order to establish whether the effect of likeability was unique to UI statements, a model including sentence type (filler vs. UI), likeability, and their interaction was fit to the data. A model containing the interaction term fared reliably better than one without (*χ2*[1] = 4.7; *β* = 0.3, *SE* = 0.14, *z* = 2.1, *p* < 0.05). This was because there were differential effects of likeability for different sentence types. Though Bobby’s likeability reliably predicted rejections to UI statements, it did not predict rejections to any other sentence type (*zs* < 0.1). Figure 2 depicts the different patterns for participants who rated Bobby highly unlikable (rated 1 or 2) versus those who rated Bobby as more likeable.

### Discussion

The results from Experiment 1 demonstrate that social context can modulate adult comprehenders’ tolerance for pragmatic violations in a binary judgment task. Findings revealed that participants in the Non-native speaker condition rated Bobby significantly less likeable than did participants in either the Unsympathetic or Sympathetic speaker condition. Moreover, participants in the Non-native speaker condition were also significantly less likely to accept UI utterances than participants in the Unsympathetic or Sympathetic speaker conditions. Finally, when collapsing across speaker conditions, results showed that participants who strongly disliked Bobby were less likely to accept critical UI items than participants who gave Bobby a higher likeability rating.

The current design does not allow us to tease apart exactly which specific social factors underlie the greater rejection rate for UI utterances in the Non-native speaker condition. It could be that participants demanded a higher threshold for correctness for non-native Bobby because he was less likeable. It could also be that they focused more on the use of quantifiers because the instructions highlighted that Bobby’s native language differs from English in this dimension. Because likeability was inversely correlated with the Non-native speaker condition, we cannot assess the independent contributions of these factors. Regardless, the results indicate that social aspects of the task influenced binary judgments for UI statements, but this was not observed for statements that were patently true or false. This pattern of results indicates that binary judgments of UI sentences are sensitive to social factors that are not directly relevant to the implicature calculation. We return to possible explanations for the surprising likeability results in the Non-native speaker condition in the General Discussion.

An unresolved question is how to interpret acceptances. Rejections of UI statements putatively indicate that an implicature was drawn, but it is not clear whether acceptances entail that no implicature was drawn. To investigate this question, we conducted an unplanned exploratory analysis of the text responses provided by participants to UI statements. If participants generated an implicature, then it would be reasonable to correct Bobby by providing a more optimally informative statement, thereby cancelling the implicature. For instance, for a UI sentence of the form “Some subsets are supersets” a participant might have provided the stronger alternative “All subsets are supersets.” Responses were coded with respect to whether they contained the stronger alternative either explicitly or using an elided form (e.g., “All of them are”). Consistent with expectations, when participants rejected UI statements, they overwhelmingly provided the stronger alternative (*M* = 85.7% of trials, *SE* = 3.1%). There were no reliable differences among speaker conditions (Sympathetic: *M* = 90.4%, *SE* = 4.3%; Unsympathetic: *M* = 82.4%, *SE* = 5.9%; Non-native: *M* = 85.2%, *SE* = 5.6%; χ2[2] = 0.9, *p* = 0.9). For acceptances, there were fewer strong alternatives provided but still a substantial number (*M* = 21.1%, *SE* = 6.1%). There was no reliable effect of speaker condition (Sympathetic: *M* = 0.8%, *SE* = 0.8%; Unsympathetic: *M* = 21.2%, *SE* = 9.0%; Non-native: *M* = 47.5%, *SE* = 16%; χ2[2] = 2.35, *p* = 0.31). It is possible that participants generated implicatures on these trials, though we cannot be certain. They may have provided the stronger statement for reasons unrelated to cancelling an unwarranted implicature. At a minimum, we can conclude that in these cases participants did not lack the cognitive resources to compute the strong alternative or to recognize its relevance to the weaker UI utterance. This indicates that participants can accept UI statements even in cases where they recognize that there are other more optimally informative utterances available.

## Experiment 2

The goal of Experiment 2 was to test whether speaker likeability continues to modulate pragmatic tolerance when participants are given a ternary rather than binary judgment task. Based on the results of Katsos and Bishop (2011), we predicted that any differences in pragmatic tolerance due to the differences in perceived speaker likeability would be reduced or eliminated. This is because the intermediate response option provides participants with an explicit way to convey that UI statements are less than optimal but are better than patently false statements. Thus most participants on most trials should choose the intermediate response option.

### Materials and Methods

#### Participants

A total of 102 English-speaking adults were recruited *via* Mechanical Turk. Eight failed to submit their data at the end of the survey, leaving data from 94 participants for analysis.

#### Materials and Procedure

The stimuli, instructions, procedure, and exit survey were identical to those in Experiment 1, with the following exception: participants were given three response options instead of two (“That’s right,” “Not quite,” “That’s wrong”).

### Results and Discussion

#### Exclusion Criteria

**Figure 3:**
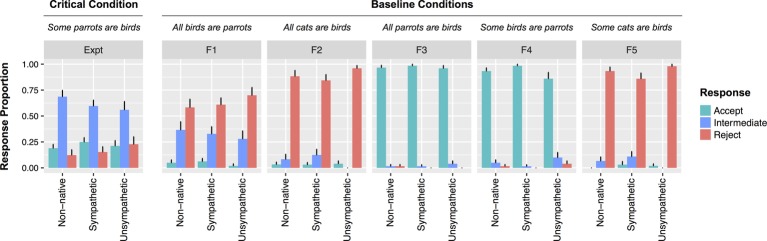
Responses to all statement types by speaker condition from Experiment 2. Error bars indicate standard errors.

Three participants were removed for indicating that they were adults when they learned English (30 or older. All other participants were 6 or younger). Filler items were judged incorrect if participants responded “That’s Right” to a patently false item (F1, F2, F5) or if they failed to respond “That’s Right” to a patently true item (F3, F4). Four participants were excluded for accuracy below 70%. The remaining participants were relatively evenly distributed across the three speaker conditions (*N_Non-native_* = 30; *N_Sympathetic_* = 32; *N_Unsympathetic_* = 25) and had high mean accuracy (*M* = 94.9%; *SE* = 0.8%) (see Figure 3).

#### Judgments of UI Statements

As predicted, the inclusion of an intermediate judgment option had clear effects on participant responses to UI items: in all speaker conditions, participants had a strong preference for the intermediate response option (Figure 3). For no other sentence type was the intermediate response the preferred option. A mixed effect model including speaker type was not reliably better at explaining the rate of rejections than one without (*χ2*[2] = 0.4, *p* = 0.82). Speaker type was also not related to the rates of acceptances (*χ2*[2] = 1, *p* = 0.61).

To establish whether speaker type had a reliably smaller effect on UI judgments in the ternary task relative to the binary task, the rejection data from both Experiments 1 and 2 were combined and fit to a model crossing experiment and speaker type. A model without the interaction of these factors fared worse than a model including the interaction (*χ2*[2] = 4.9, *p* = 0.087). Thus speaker type had a stronger effect for the binary judgment task relative to the ternary judgment task on rejection rates. To investigate this interaction further, models were fit to subsets of the data consisting of each pair of the three speaker conditions. The difference between rejection rates in the Non-native and Unsympathetic speaker conditions was reliably different across experiments (*β* = 2.05, *SE* = 0.95, *z* = 2.17, *p* < 0.05). For the Non-native and Sympathetic conditions, this difference was marginally reliable across experiments (*β* = 1.5, *SE* = 0.84, *z* = 1.83, *p* = 0.067). In contrast, there was no interaction between speaker type and experiment in predicting rejection rates for the Sympathetic and Unsympathetic speaker conditions. (*z* = 0.59).

For acceptances, a model containing the interaction of experiment and speaker type was numerically, but not reliably, better at explaining the data than one without (*χ2*[2] = 2.8, *p* = 0.25). When considering just the Non-native and Unsympathetic speaker conditions, there was a marginal interaction between speaker type and experiment (*β* = 0.92, *SE* = 0.54, *z* = 1.7, *p* = 0.09). This arose because speaker type had a stronger effect on acceptances for the binary judgment task than for the ternary judgment task. There was no interaction in acceptances between the Non-native and Sympathetic speaker conditions across experiments (*z* = 0.93). Nor was there an interaction in acceptance rates for the Unsympathetic and Sympathetic speaker conditions across experiments (*z* = 0.74).

#### Exit Survey

Accuracy patterns in Experiment 2 were similar to those from Experiment 1. Participants were extremely accurate at providing Bobby’s age (91.2%), and academic subject (97.8%). Performance did not differ across conditions (*t*s < 1). Performance was again worse for remembering the critical description of Bobby (85.7%). Participants in the Sympathetic and Non-native conditions were highly accurate (94.1 and 100% respectively), but participants in the Unsympathetic Bobby condition were much less accurate (57.7%). When only data from participants who described Bobby correctly were included in the analysis of rejection rates, the pattern was similar to results from all participants. Speaker condition did not reliably predict rejections (*χ2*[2] = −0.74, *p* = 1), acceptances (*χ2*[2] = 0.57, *p* = 0.75), or intermediate responses (*χ2*[2] = 1.11, *p* = 0.57).

When including just those participants who correctly recalled the speaker description, the interactions across experiments in rejection and acceptance rates became more apparent. A model containing the interaction of experiment and speaker type on rejections performed marginally better than a model without this term (*χ2*[2] = 4.75, *p* = 0.09). There was an interaction between speaker type and experiment rejection rates reliable for the Non-native and Sympathetic speaker conditions (*β* = 1.83, *SE* = 0.89, *z* = 2.07, *p* < 0.05) and marginal for the Non-native and Unsympathetic conditions (*β* = 2.16, *SE* = 1.26, *z* = 1.7, *p* = 0.08). There was no such interaction for the Sympathetic and Unsympathetic speaker conditions (*z* = 0.01). Thus, the effect of speaker type on rejection rates was reliably larger for Experiment 1 with binary response options compared to Experiment 2 with ternary response options.

Parallel analyses were performed on acceptances using only data from participants who described Bobby correctly. A model containing the interaction between speaker type and experiment was marginally better than a model that did not contain this term (*χ2*[2] = 4.9, *p* = 0.08). There was an interaction between speaker type and experiment when considering just the Non-native and Unsympathetic speaker conditions (*β* = 1.32, *SE* = 0.63, *z* = 2.11, *p* < 0.05). There was a trend toward an interaction for the Non-native and Sympathetic speaker conditions across experiments (*β* = 0.72, *SE* = 0.53, *z* = 1.35, *p* = 0.18). There was no interaction across experiments for the Unsympathetic and Sympathetic speaker conditions (*z* = 0.81). Thus, just as with rejections, the effect of speaker type on acceptances was larger with binary response options than with ternary response options.

Experiment 2 also replicated the surprising speaker-likeability finding from Experiment 1: participants in the Non-native speaker condition rated Bobby significantly less likeable than did participants in the Unsympathetic or Sympathetic conditions (Non-native: *M* = 2.10, *SE* = 0.23; Unsympathetic: *M* = 5.92, *SE* = 0.23; Sympathetic: *M* = 5.76, *SE* = 0.20). Exit survey results also revealed that only 58% of participants in the Unsympathetic condition selected “obnoxious” as the best description of Bobby, while the remaining 42% selected “kind.” In contrast, 95 and 100% of participants in the Sympathetic and Non-native speaker conditions respectively selected the appropriate descriptor (see General Discussion for possible explanations for this finding).

**Figure 4:**
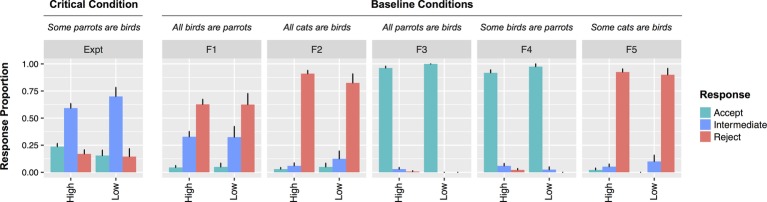
Responses by Low (1–2) vs. High (3–7) speaker likeability from Experiment 2. Note that all analyses in the text were performed with likeability as a (non-dichotomized) continuous variable.

However, in contrast to the results found with the binary judgment task in Experiment 1, likeability had no effect on rejections (*χ2*[2] = 1.08, *p* = 0.3). There was also no relationship between likeability and acceptances (*χ2*[2] = 0.17, *p* = 0.68). When only participants who accurately recalled the description of Bobby were included, these patterns were unchanged (rejections: *χ2*[2] = 0.58, *p* = 0.44; acceptances: *χ2*[2] = 0.9, *p* = 0.34). To investigate whether the effect of likeability was different for UI and other sentence types (filler vs. UI) an interactional analysis was performed. There was a main effect of sentence type whereby fillers were rejected more often than UI statements (*β* = 0.99, *SE* = 0.24, *z* = 4.06, *p* < 0.001). There was no effect of likeability (*z =* 0.88, *p* = 0.38). Importantly, there was no interaction between likeability and sentence type in predicting rejections (*z* = 0.91, *p* = 0.36) nor acceptances (*z* = 0.92, *p* = 0.36). Thus, unlike Experiment 1 where responses to UI items were specifically affected by likeability for binary judgments, there was no difference in the (null) effects of likeability for ternary judgments (See Figure 4).

### Discussion

The results from Experiment 2 indicate that social context did not modulate participants’ tolerance for pragmatic violations when participants were given an intermediate option in a ternary judgment task. In contrast to Experiment 1, the positive correlation between speaker likeability and acceptance of critical items is eliminated when participants have an intermediate response option. This indicates that the locus of social context effects in Experiment 1 was in selecting a response (i.e., determining what the threshold for rejection is), rather than being related to computing the inference.

Experiment 2 also addresses a potential concern with the speaker manipulation in Experiment 1. Though the task itself is unchanged across speaker conditions, it is still logically possible that the manipulation of speaker description somehow affected implicature calculation indirectly. For instance, if the speaker descriptions fundamentally changed the communicative goals of the task in disparate ways. If so, then it is conceivable that the results from Experiment 1 reflect differences in implicature rates across conditions rather than differences in response selection. The results from Experiment 2 rebut this interpretation. The rates of implicatures in Experiment 2 (inferred from either rejections or acceptances) were not affected by speaker condition nor likeability as they were in Experiment 1. Since the only difference between Experiments 1 and 2 is the response options available to participants, this difference strongly indicates that implicature processes were unaffected.

Similar exploratory analyses to those in Experiment 1 were performed on participants’ text feedback. The rate of strong alternative statements provided for trials in which the participant did not accept the UI statement (both intermediate responses and rejections) was similar to Experiment 1 (*M* = 85.0%, *SE* = 3.1%). There were no reliable differences among speaker conditions (Sympathetic: *M* = 82.8%, *SE* = 5.5%; Unsympathetic: *M* = 80.5%, *SE* = 7.6%; Non-native: *M* = 91.1%, *SE* = 4.3%; χ2[2] = 2.3, *p* = 0.32). For trials on which the participant accepted the UI statement, the rate of feedback containing strong alternative statements (*M* = 3.8%, *SE* = 2.8%) was numerically lower than that for acceptances in Experiment 1 (*M* = 21.1%). There were no reliable differences for different speaker conditions (Sympathetic: *M* = 3.4%, *SE* = 2.6%; Unsympathetic: *M* = 0%, Non-native: *M* = 7.1%, *SE* = 7.1%; model unidentifiable). One possible explanation for the reduction from Experiment 1 to 2 is that participants who generated an implicature and who wanted to provide corrective feedback for Bobby without rejecting his statement could avail themselves of the intermediate response in Experiment 2. In Experiment 1, they would have had to accept the statement.

## General Discussion

We set out to test whether manipulating social context can modulate adult acceptability judgments of UI utterances. We manipulated the perceived likeability of the speaker by providing participants with a specific social context and a detailed description of their interlocutor against which they were asked to make their judgments. In Experiment 1, participants rejected UI utterances from the Non-native speaker more frequently than from either the Unsympathetic or Sympathetic speakers when given only a binary response option. At the same time, participants disliked the Non-native speaker relative to the other speakers. This pattern of effects indicates that social context can influence pragmatic judgments when participants are forced to choose between rejection and acceptance. Note that the cognitive task was identical in all conditions and participants were randomly assigned to speaker conditions. Thus, it is unlikely that participants in the Non-native speaker condition had more cognitive resources than those in the other conditions. Moreover, participants were equally accurate on filler items across conditions. Social factors only influenced judgments on the UI items, where the pragmatic and literal meanings diverged.

In Experiment 2, the same materials were employed, but participants had three response options and could therefore give more graded feedback. In this case, the acceptance rate was not affected by our social context manipulation. Thus, the positive correlation between speaker likeability and acceptance of critical items is eliminated when participants have an intermediate response option. In this case, participants did not have to deliberate over where to place the boundary of acceptability—the intermediate response option provided participants with an explicit way to signal that UI statements are less than optimal but are better than patently false statements.

The relative likeability of the speakers is somewhat surprising. We had predicted that the Unsympathetic speaker condition would engender the least amount of charitability from participants. However, both experiments found that likeability ratings were lowest in the Non-native speaker condition. One possible explanation for this unexpected result is that participants were displaying ethnocentric tendencies (were prejudiced against non-native speakers and/or immigrants). An alternative explanation may be related to the high rate of patently false statements (30% of the items) in the experimental design. Participants may have been able to rationalize such “poor performance” from both the Sympathetic and Unsympathetic speakers: Sympathetic Bobby was described as having a developmental disorder and Unsympathetic Bobby was described as “very difficult.” Non-native Bobby, on the other hand, was described as “bright.” This may have led participants in the Non-native speaker condition to become more irritated with his poor performance. A related finding was also surprising. Likeability ratings for Unsympathetic Bobby were not reliably different than ratings for Sympathetic Bobby (even among participants who correctly remembered unsympathetic Bobby being labeled “obnoxious” by his teachers). One possible explanation for this finding is that Unsympathetic Bobby may have garnered compassion rather than aversion; participants may have attributed his poor behavior to external causes (e.g., poor parenting) rather than to the child himself. Importantly, these issues are tangential to the critical finding. Because these manipulations should not directly influence the actual computation of a scalar inference, any difference in responses between binary and ternary judgments is better explained by differences in response selection processes than by different rates of implicature computation. Therefore, we take the current findings as clear evidence that social factors unrelated to generating the implicature itself can modulate adult comprehenders’ tolerance for pragmatic violations in a binary judgment task.

An open question is how to interpret acceptances in the present studies. One possibility is that participants in Experiment 1 recognized that Bobby’s utterance was not optimally informative, but decided that this violation was not sufficient to assign it the same rating as patently false statements. If so, many of these individuals would have likely preferred an intermediate option. On this view, we should have seen a reduction in the rate of acceptances in Experiment 2. There were indeed small numerical reductions for the Unsympathetic speaker (30.6 vs. 22.9%) and for the Sympathetic speaker (27.5 vs. 24%) who were both deemed likeable, but the rate of acceptances increased slightly for the Non-native speakers (13.3 vs. 19%) who were deemed unlikeable. However, the overall rate of acceptances did not fall dramatically when provided with an intermediate option. There are at least two plausible accounts for this. One is that there were, by chance, fewer genuine implicatures drawn in Experiment 2 than Experiment 1. On this view, non-acceptances in the ternary task might more accurately reflect implicature generation than rejections in the binary task. If so, then the small reduction in acceptances from Experiment 1 to Experiment 2 would have been larger if the two groups of participants generated implicatures at the same rate. A second possibility is that the intermediate responses were still too harsh for some individuals who generated implicatures. As a result, they elected to accept UI statements even with an intermediate option available. In this case, an additional intermediate option (e.g., “mostly right”) might have revealed still more individuals who are sensitive to underinformativity (see Jasbi, Waldon, and Degen, submitted). Either of these possibilities, either singly or in combination, could have led to the pattern observed.

## Conclusion

The present studies demonstrate that pragmatic tolerance can contribute to the variability found in adult responses to UI utterances in binary judgment tasks. Many studies take the non-acceptance of a UI statement to be evidence that the comprehender has computed a scalar inference and the acceptance of a UI statement as evidence that they have not. The results above call these assumptions into question. We have shown that adult comprehenders, like children (Katsos and Bishop, 2011), will accept a UI statement even in the same situations where they recognize it as non-optimal. Unlike patently false or true statements, UIs are neither completely wrong nor completely correct. When forced to select between two inapt options in a binary choice task, social factors can tip the balance so that participants choose to reject UI statements more often for certain speakers. In contrast, a ternary judgment task allows participants to clearly indicate that UI utterances are intermediately acceptable between patently true and false statements. With a more apt intermediate response option, participants are not as affected by social aspects of the speaker. More work is needed to establish what aspects of the social context are most influential for binary judgments, and to determine why children are less likely to reject pragmatically infelicitous statements than adults.

What we do have evidence for is that binary judgments are affected by selection processes, which are unrelated to implicature computation, in a way that graded judgments are not. Binary judgments are perhaps the most widespread method for investigating implicature processing and development. The present work thus demonstrates that results garnered from binary judgment tasks must be interpreted with caution.

## Data Availability

The data sets analyzed for this study can be found at https://drive.google.com/drive/folders/15qSxN7dXPP7GKKJA8Ks9dr3nInJf4OL6?usp=sharing

## Ethics Statement

This study was carried out in accordance with the recommendations set forth in the Belmont Report, federal regulations (e.g., DHHS regulations 45 CFR Part 46), and Swarthmore College policies. The protocol was approved by the Swarthmore College Internal Review Board. All subjects gave written informed consent in accordance with the Declaration of Helsinki. The protocol was approved by the Internal Review Board at Swarthmore College. Participants were provided with and completed an electronic consent form before beginning the study.

## Author Contributions

DG, LS, and MK all made contributions to the conception and design, study implementation, data acquisition an analysis, interpretation, and write up of this work.

### Conflict of Interest Statement

The authors declare that the research was conducted in the absence of any commercial or financial relationships that could be construed as a potential conflict of interest.

## Footnotes

^1^This is true regardless of whether the task is a statement evaluation task or a truth value judgment (see Foppolo et al., 2012).
